# Lessons from a Flash Flood in Tehran Subway, Iran

**DOI:** 10.1371/currents.dis.b80efb4a82b7bc2b9dbb5280d79da497

**Published:** 2013-05-02

**Authors:** Abbas Ostad Taghizadeh, Seyedeh Vahideh Soleimani, Ali Ardalan

**Affiliations:** Department of Disaster Public Health, School of Public Health, Tehran University of Medical Sciences, Tehran, Iran; Department of Disaster & Emergency Health, National Institute of Health Research, Tehran University of Medical Sciences, Tehran, Iran; Department of Disaster Public Health, School of Public Health, Tehran University of Medical Sciences, Tehran, Iran

## Brief Incident Report

On 15 April 2012 at around 13:00 local time, following a heavy rainfall in Tehran, a break in the channel wall of Kan River caused a flash flood in the Tehran metro tunnels. Consequently, line 4 of the metro went out of operation for about two weeks 1 . The flood was running for 30 hours in the metro system so that three stations were flooded causing damage to four trains and 28 wagons 2. It is estimated that approximately one million m^3^ of water and debris rushed in the metro tunnels through the break. Although this flash flood caused property damage of about $21 million (at the official exchange rate) to the metro especially its electrical system, fortunately no death and injury ensued 1 ^,^ 2 ^,^ 3 ^,^ 4 .

Quick measures were taken to disconnect electricity and evacuate more than 1,500 passengers and staff from the adjacent stations while simultaneously the water was draining. For a few days, more than 10 thousands m^3^ of debris and precipitants were removed from the stations and trains. Finally, damaged stations were repaired and got back to the operation in 12 days 3 . It was claimed that 2,500 persons worked every day during the response to this disaster.

Immediate disconnection of the electricity system prevented electrocutions. Timely emergency evacuation in this event saved hundreds of lives. All trains were stopped in adjacent stations and the passengers were instructed by the staff to safely evacuate the metro. In addition, dwellers near at risk stations were relocated to safe buildings.

Here, we wish to address some challenges along with corresponding lessons learned that have a bearing on revisions to the emergency plan of Tehran in general and Tehran’s metro in particular:

1. Experts believe that over excavation near the river channel was the main reason for this event 1 . In fact, millions of dollar could have been saved if excavation code was respected. Rigorous supervision of construction activities in urban areas will prevent similar events in the future.
2. Tehran’s early warning system (EWS) for flash floods must be improved. City’s inhabitants and the infrastructures including Tehran’s metro, in particular, will benefit from this system. A timely and effective EWS prevents the loss of lives and unnecessary functional and economical damages. It however requires further collaboration among Tehran’s Disaster Management Center, Meteorological Organization and Water and Sanitation Organization.
3. Risk transfer is a key component of disaster risk management. Tehran's metro system was insured up to $8 million (at the official exchange rate), but the damage caused by the flash flood far exceeded that amount 2 . This challenge could be prevented through more detailed risk assessment.
4. Tehran’s metro needs a paratransit system in case of major emergencies. This system will be also useful in case of daily incidents. Once the metro service was disrupted, a large crowd of passengers was wandering in the streets looking for taxies.
5. While fortunately no death was reported in this event, but it is worth emphasizing continuous education of metro’s passengers in term of safety issues especially risks of electrocution during the flooding and stampede toward the exit doors. This will save lives and assist the metro operators for safer and quicker response.

## Funding Statement

The authors received no fund for this study.

## Competing Interests

The authors have declared that no competing interests exist.

## Corresponding Author

Ali Ardalan MD, PhD. Tehran University of Medical Sciences. Harvard Humanitarian Initiative. Email: ardalan@hsph.harvard.edu
